# Eosinophilic oesophagitis in a Nigerian adolescent: a case report

**DOI:** 10.11604/pamj.2024.47.3.36280

**Published:** 2024-01-04

**Authors:** Ikobah Joanah Moses, Ikwuagwu Elekwachi, Ita Ukpabio, Theophilus Ugbem, Omeodu Chimankpan Okechukwu, Emmanuel Ekanem

**Affiliations:** 1 Paediatrics Gastroenterology, Hepatology and Nutrition Unit, Department of Paediatrics, University of Calabar, Calabar, Cross River State, Nigeria,; 2 Department of Paediatrics, University of Calabar Teaching Hospital, Calabar, Cross River State, Nigeria,; 3 Department of Anaesthesiology, University of Calabar Teaching Hospital, Calabar, Cross River State, Nigeria,; 4 Department of Pathology, University of Calabar, Calabar, Cross River State, Nigeria

**Keywords:** Eosinophilic oesophagitis, eosinophils, gastro-oesophageal reflux disease, oesophagogastroduodenoscopy, case report

## Abstract

Eosinophilic oesophagitis (EoE) is a chronic immune and antigen-mediated disease characterized by symptoms related to oesophageal dysfunction, and histologically, is marked by eosinophilic infiltrate in the oesophageal mucosa. It is prevalent in developed countries and considered rare in developing countries. There is an interplay of allergic and genetic factors in the aetiology of EoE. This is a report of EoE in a 15-year-old female adolescent in Nigeria who presented to the University of Calabar Teaching Hospital with recurrent vomiting, abdominal pain, weight loss, and dysphagia. She had received treatment for Gastro-oesophageal disease three years earlier and was lost to follow-up. Weight on admission was 39 kg and height 170 cm with a BMI below the 3^rd^ centile. Peripheral blood showed an eosinophil count of four percent. The abdominal computed tomography (CT) scan and upper gastrointestinal (GI) series were normal. Faecal antigen for H. pylori and ova for stool parasites were negative. Histologic findings of proximal and distal oesophageal mucosal biopsies showed greater than 20 eosinophils per high power field. The histology of the stomach and duodenum were normal. She was initially treated with a protein pump inhibitor, with no improvement. Swallowed fluticasone propionate and eliminating peanuts, wheat, egg, and milk from her diet were introduced. Symptoms improved with the patient no longer vomiting and had an increase in weight gain. She was discharged to follow up. This case shows that EoE occurs in developing countries, but diagnosis may be missed. There is a need for a high index of suspicion among gastroenterologists in patients with symptoms suggestive of GERD not responding to therapy.

## Introduction

Eosinophilic oesophagitis (EoE) is a chronic allergen and immune-mediated disease manifesting with oesophageal dysfunction and infiltration of oesophageal mucosa with eosinophils in the absence of secondary causes of eosinophilia [1]. Eosinophilic oesophagitis was first described as a distinct clinicopathological disease in the 1990s and was previously considered a rare disease [2]. In recent years, incidence and prevalence rates have increased significantly and it is now a common cause of upper gastrointestinal tract morbidity [2]. A recent meta-analysis calculated pooled incidence and prevalence rates in children of 5.1/100,000/year and 29.5/100,000 respectively [1]. These rates vary widely with location, being higher in Western countries and lower in Eastern countries [1]. Eosinophilic oesophagitis affects all age groups and is more common in Caucasian males [2]. The aetiology of EoE is believed to have a genetic component that acts in the context of environmental exposures to increase the risk of developing the disease [3]. Risk factors implicated in EoE include Caesarian delivery, preterm delivery, use of antibiotics in infancy, history of allergic rhinitis and allergic dermatitis [1]. The pathogenesis of EoE is not completely understood [2]. It is an allergic disease activated by food and environmental allergens through IgE- and non-IgE-mediated mechanisms [4]. The clinical manifestations of EoE are diverse and vary widely based on age [2,5]. Young children have non-specific symptoms including vomiting, abdominal pain, feeding difficulties such as prolonged mealtime and failure to thrive [2]. Adolescents and adults typically have dysphagia and food impaction [5]. Untreated EoE could lead to complications including the development of oesophageal strictures, narrowing of the oesophagus and formation of rings [2]. Eosinophilic oesophagitis, though found globally, is believed to be uncommon in developing countries and is scarcely reported from sub-Saharan Africa and India [1,6]. We report a case of EoE in Nigeria, diagnosed in the paediatric age group at the University of Calabar Teaching Hospital, Calabar.

## Patient and observation

**Patient information:** a 15-year-old female Nigerian adolescent presented to the University of Calabar Teaching Hospital, Calabar, Cross River State, Nigeria with recurrent vomiting, abdominal pain, frequent regurgitation, bloating, dysphagia, and weight loss of three months duration. Vomiting was triggered by feeds and vomited an average of five to six times a day. Vomitus was non-bloody, non-bilious, and consisted of undigested food. Abdominal pain was localized around the epigastrium. Dysphagia was insidious in onset and gradually worsened until she could tolerate only liquid meals. There was a family history of atopy. Peanuts, milk, fish, eggs and wheat-based foods worsened her symptoms.

**Clinical findings:** she was ill-looking, wasted, and mildly pale. Her weight was 39kg (on the 0-z score weight for age), height 1.71 metres (>+1 z score height for age) and BMI 13.4kg/m^2^ (<-3 z score for age). Abdominal examination revealed mild epigastric tenderness with no organomegaly. Examination of other systems was essentially normal.

**Timeline:** she was first seen at the Paediatric Gastroenterology Clinic three years before this presentation, with a two-year history of recurrent vomiting and epigastric pain. An oesophagogastroduodenoscopy done showed normal oesophageal mucosa, but biopsies were not taken due to financial constraints. She was treated for gastroesophageal reflux disease (GERD) with an oral proton pump inhibitor and lifestyle modification. She experienced some clinical improvement. However, she defaulted to follow-up until she re-presented with worsening clinical symptoms three years later.

**Figure 1:**
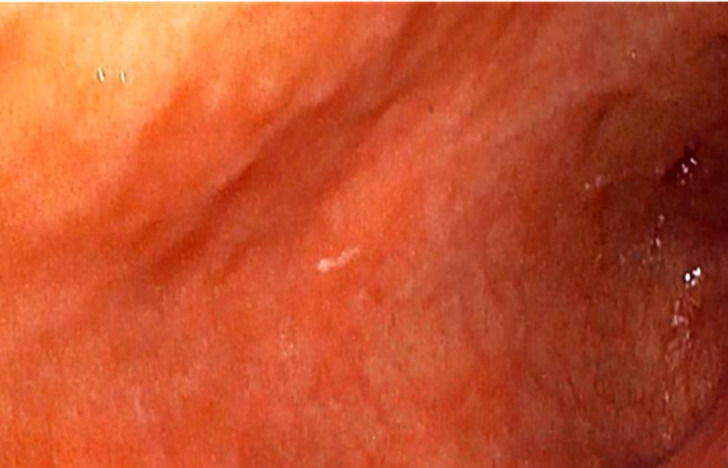
endoscopic view of the normal oesophagus

**Figure 2:**
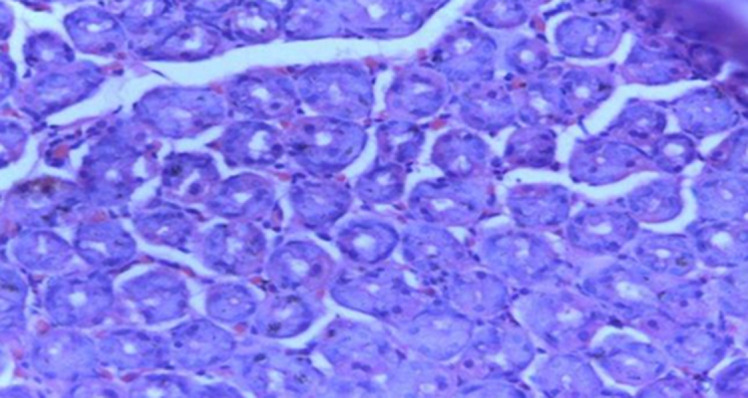
proximal oesophageal biopsy showing heavy eosinophilic infiltration > 30 eosinophils per high power field using H and E stain before treatment

**Diagnostic assessment:** her packed cell volume was 29%, red blood cells appeared microcytic and hypochromic, and erythrocyte sedimentation rate was 10mm/hr (Westergren method). Radiological studies, including abdominal ultrasound, upper gastrointestinal contrast studies and contrast computed tomography scan of the abdomen were all normal. Stool microscopy showed no ova or parasites seen. Faecal Antigen for *H. pylori* test was negative. Biochemical and serological tests for HIV, HBsAg and HCV were negative. Oesophagogastroduodenoscopy done at this time showed normal mucosa of the oesophagus (Figure 1). Histologic examination of oesophageal biopsy specimens from the proximal and distal oesophagus showed a marked infiltration of >20 eosinophils/high power field confirming a diagnosis of EoE. This is shown in Figure 2. Histology of biopsies of the stomach and duodenum were normal.

**Therapeutic intervention:** she was admitted into the paediatric medical ward on account of the severity of her symptoms and was treated with proton pump inhibitor (PPI) while awaiting the histologic result of the oesophageal biopsy. Without improvement of symptoms, oral corticosteroid was commenced with findings of oesophageal biopsy in keeping with EoE. Swallowed fluticasone propionate was commenced with significant improvement in symptoms. Peanuts, milk, fish, eggs, and wheat were eliminated from her diet. Symptoms resolved, but she gained weight and was discharged for follow-up.

**Figure 3:**
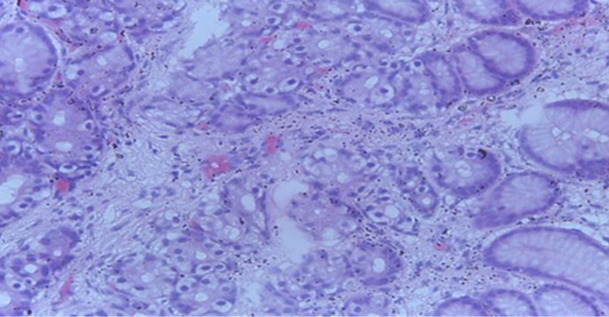
proximal oesophageal mucosa biopsy showing reduced eosinophilic infiltration < 15 eosinophils per high power field using H and E stain after treatment with swallowed fluticasone propionate

**Follow-up and outcome:** patient was consistent with follow-up visits. She remained stable on the elimination diet and use of swallowed steroids. However, a repeat oesophagogastroduo denoscopy with biopsies was delayed to 6-month during the follow-up period due to financial constraints. Findings of reduction in the eosinophil/high power field count to below 15 in biopsies taken from the proximal and distal oesophagus at the 6-month follow-up biopsy are shown in Figure 3. Her blood-packed cell volume had normalized at 36% with normochromic, normocytic appearance of red blood cells. The patient had no adverse reactions to prescribed medications and is adhering to treatment.

**Patient perspective:** the patient and her parents were satisfied with the timely therapeutic intervention offered and the positive response to the appropriate diagnosis and treatment made available. She is thankful for the care that all the doctors and nurses show her.

**Informed consent:** it has been obtained from the patient.

## Discussion

This report describes the first case of EoE in a Nigerian adolescent. The diagnosis was made based on clinical presentation and upper gastrointestinal endoscopy with biopsies taken from the proximal and distal oesophagus showing >20 eosinophils per high power field. Histologic findings of biopsies of the stomach and duodenum were normal. Eosinophilic oesophagitis is uncommon in sub-Saharan Africa [1]. However, the disease is under-studied in this region [5] so there is insufficient data to ascertain the true epidemiology of the disease in the region. This paucity of data may be due to the lack of widespread availability of paediatric gastroenterologists or facilities for endoscopy. In Nigeria, paediatric endoscopy started recently [7]. Eosinophilic oesophagitis may be misdiagnosed in some patients due to a lack of awareness of its existence and its similarity with more common gastrointestinal disorders such as GERD. The non-specific clinical presentation of EoE and the similarity to diseases like GERD frequently lead to delays in diagnosis [2]. In our patient, as in most cases of EoE reported in the literature [5], the diagnosis was delayed by five years. The prolonged delay in our patient was due to financial constraints from parents as medical services in this locale are mainly funded by out-of-pocket payments and loss to follow-up could still have been due to financial constraints. A high index of suspicion is therefore required to make the diagnosis. The average delay from onset of symptoms to diagnosis reported in the literature ranges from 1.2 to 3.5 years in children [5].

Several risk factors for EoE have been identified and could enhance clinical suspicion of EoE [6]. Environmental risk factors associated with EoE include early life exposures such as maternal fever during pregnancy, caesarian delivery, prematurity, antibiotic exposure during infancy, and lack of early exposure to microbes [3,6,8] *H. pylori* infection seems to have a protective effect [1,6]. Our patient had a negative test result for faecal Ag for *H. pylori*. Genetic risk factors include the presence of a family member with EoE, as well as a personal or family history of atopy [3]. Up to 75% of individuals with EoE have a personal or family history of atopic diseases such as allergic rhinitis, asthma, or food allergies [3]. Our patient had a personal and family history of atopy.

The diagnosis of EoE requires the presence of clinical manifestations, histologic findings in oesophageal mucosa and exclusion of other causes of oesophageal eosinophilia [6]. The most reported symptoms in children are vomiting, abdominal pain, and dysphagia [5]. Our patient presented with all three symptoms. The gold standard investigation for diagnosis of EoE is upper GI endoscopy with biopsies taken from the oesophagus, stomach, and duodenum [9]. Typical endoscopic features in children include exudates, mucosal oedema, whitish furrows, basal hyperplasia, formation of rings or strictures [6,10]. The oesophageal mucosa may be normal in up to 25% of cases [10]. In our patient, the oesophagus appeared normal. It is therefore necessary to obtain biopsies from patients in whom there is a clinical suspicion of EoE even if there are no abnormalities of the oesophageal mucosa on endoscopy [10]. It is recommended that multiple biopsies should be taken from the proximal and distal oesophagus to maximize the sensitivity of the histologic report due to the patchy eosinophilic infiltration of the oesophagus [10]. Additionally, biopsies from the stomach and duodenum are also recommended to rule out other diseases such as eosinophilic gastroenteritis [10]. Our patient had multiple biopsies taken from the proximal and distal oesophagus as well as the stomach and duodenum. The histologic hallmark of EoE is the presence of > 15 eosinophils per high power field in the oesophageal epithelium [9,10] like findings in our patient (Figure 2). Oesophageal eosinophilia is key to the diagnosis of EoE [1]. However, it is important to rule out secondary causes of oesophageal eosinophilia by taking biopsies from the stomach and duodenum which usually give normal findings in the diagnosis of EoE [1,2,6].

Recent consensus guidelines recommend PPIs, corticosteroids, and elimination diets as first-line options for the treatment of EoE in children [9]. Proton pump inhibitors are effective in inducing and maintaining remission and are thought to have anti-inflammatory properties. They are recommended as the first-choice treatment in EoE because of their low cost, safety profile and ease of administration [9,10]. Topical and systemic corticosteroids are equally effective in inducing and maintaining remission in EoE [9]. Systemic corticosteroids induce remission quickly and are useful in patients with severe symptoms including severe dysphagia and weight loss [6]. However, their use is limited by significant adverse effects with prolonged use [9]. Topical corticosteroids such as swallowed fluticasone propionate and oral viscous budesonide are the mainstay of pharmacotherapy in EoE because of their effectiveness and limited side effects [2]. Our patient initially received PPI and oral prednisolone due to the severe dysphagia and marked weight loss. She was later transitioned to swallowed fluticasone propionate. Dietary strategies for the treatment of EoE include elemental diets, empiric elimination diets and targeted (testing-directed) elimination diets. Elemental diets have significant drawbacks that limit clinical use while targeted elimination diets are poorly effective and not recommended [10]. The empiric elimination diet based on the elimination of the most common foods known to trigger EoE (milk, wheat, egg, soy, nuts, and fish/shellfish) is the preferred dietary approach to the management of EoE [10]. Wheat and peanuts were eliminated from our patient´s diet and this resulted in reduced frequency of vomiting.

The resolution of symptoms after treatment for EoE is not a reliable indicator of histologic remission because patient-reported symptoms have been found not to correlate well with histologic activity [10]. Therefore, endoscopy with biopsy should be repeated to confirm histologic remission at least 6-16 weeks after initiation of treatment [10]. A repeat endoscopy for our patient was delayed until the six-month follow-up due to financial constraints. Biopsy samples obtained showed <15 eosinophils per high power field thus confirming histologic remission.

## Conclusion

This case report shows that EoE occurs in Nigerian children and can be successfully managed using available resources. Increased awareness of the disease, and improved access to Paediatric gastroenterologists, and facilities for endoscopy would throw more light on the true meaning of the disease among children in Nigeria.
